# Effect of Repeated Greater Occipital Nerve Block in Patients with Ocular Neuropathic Pain: A Retrospective Observational Study

**DOI:** 10.3390/jcm12237454

**Published:** 2023-12-01

**Authors:** Jonghwan Lee, Woochan Park, Jinyoung Choi, Geonho Lee, Seokhyun Ma, Seungcheol Lee, Sangyoong Park

**Affiliations:** 1 Department of Anesthesiology and Pain Medicine, Dong-A University Hospital, 26 Daesingongwon-ro, Seo-gu, Busan 49201, Republic of Korea; 2 Department of Ophthalmology, Dong-A University Hospital, 26 Daesingongwon-ro, Seo-gu, Busan 49201, Republic of Korea

**Keywords:** convergence, greater occipital nerve block, ocular neuropathic pain, pain relief scale, trigeminocervical complex

## Abstract

Ocular neuropathic pain (ONP) has various etiologies, and patients have various symptoms. The clinical management of patients with ONP has been debated. We aimed to evaluate the effect of repeated greater occipital nerve block (GONB) on ONP based on convergence in the trigeminocervical complex. In this single-center retrospective study, the medical records of 204 patients who were referred to the pain clinic by the ophthalmology department of our hospital and subsequently underwent repeated GONB for chronic ONP between January 2008 and February 2022 were analyzed. They received GONB every two weeks, up to 10 times. Symptoms of ONP were divided into five categories: eye pain, dysesthesias/allodynia, non-eye pain, visual disturbance, and tearing. The primary outcome of this study was the pain relief scale (PRS) score after repeated injections. The differences and magnitude of decrease in the overall pain relief scale score were statistically significant (estimate = −0.55, *p* < 0.001). There were two patients who had recurrence of ONP and seven patients who had adverse events. According to our study, repeated GONB can reduce symptom severity in patients with ONP. Therefore, it appears that GONB can be considered a multimodal management method for ONP.

## 1. Introduction

Some have defined ocular neuropathic pain (ONP) as ocular pain caused by a primary lesion or dysfunction of the somatosensory system [1]. ONP is also termed ocular neuralgia, keratoneuralgia, or ocular neuropathy. Based on its definition, ONP can be caused by an ocular surface lesion that has been identified via slit lamp. However, in some cases, the severity of symptoms is not proportional to the severity of the lesion [2]. With neuropathic pain, minimal to no signs can be observed through a slit lamp by ophthalmologists. This indicates that ONP can present without any ophthalmologic findings, making it extremely difficult to identify [1,2]. The disparity between signs and symptoms often results in patients being dismissed or considered malingering, hysterical, or psychosomatic. Because of the difficulty of explaining the disease, ONP has not been accurately defined. Although the exact epidemiology of the disease is not yet explicated, an increased number of patients with ONP may describe various symptoms, such as pain, discomfort, dryness, irritation, grittiness, blurred vision, and photoallodynia [2]. The symptoms may substantially affect the quality of life of the patients and may cause impaired functioning in terms of performing activities of daily living [3].

**Figure 1:**
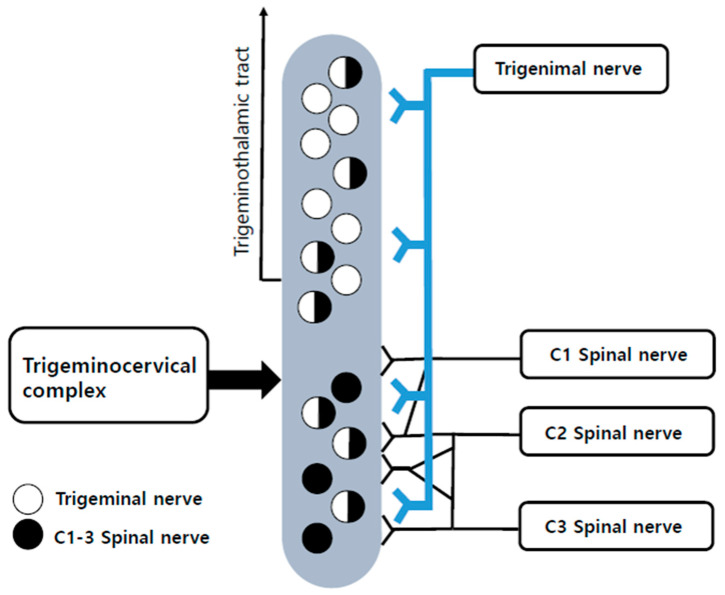
Anatomic and physiologic distribution of the elements involved in trigeminocervical convergence. C1, first cervical spine; C2, second cervical spine; C3, third cervical spine; empty circle, secondary neurons receiving inputs from the trigeminal nerve; full-colored circle, secondary neurons receiving inputs from the upper cervical nerve; half-colored circle, secondary neurons receiving inputs from the upper cervical and the trigeminal nerve.

The pathophysiology of ONP is related to the complex interplay between the central and peripheral nervous systems but remains unclear [2]. Ocular inflammatory diseases, including dry eye, keratitis, conjunctivitis, corneal ulcer, neoplasm, surgical interventions, or trauma, can be the underlying cause of ocular neuropathic pain [2,4,5]. Some systemic diseases, including Sjögren’s syndrome, fibromyalgia, diabetes, and depression, are associated with ONP [5]. Although the mechanism of ONP is unclear, peripheral and central sensitization are considered to play important roles in the pathogenesis of ONP. The structures surrounding the eye, including the cornea, eyelid, and conjunctiva, are innervated by branches of the ophthalmic nerve, a division of the trigeminal nerve. Multiple studies have reported that occipital neuralgia can be described in relation to structures innervated by the branches of the trigeminal nerve, namely, the forehead, temples, and eyes [6,7,8]. Additionally, multiple clinical studies and case reports have confirmed that pain originating in the neck can be referred to the eye [7,8,9]. Ocular nociceptors have their primary cell bodies in the trigeminal ganglion and synapse in the trigeminal subnucleus caudalis/upper cervical transition zone, which converges and forms the trigeminocervical complex (TCC) (Figure 1) [10]. Second-order axons from the complex relay information to the thalamus via the spinothalamic pathway. In the TCC, the branches of the afferent trigeminal nerve converge at the same body cluster as afferents from the upper cervical spinal nerve. The second cervical spinal nerve (C2) and third cervical spinal nerve (C3) segments that make up the occipital nerves share a relay complex with the trigeminal cell bodies [6]. Cells inside these nuclei are considered multimodal neurons. The trigeminal nucleus caudalis (TNC) also receives afferents from the autonomic nervous system (vagus nerve) and the hypoglossal nerve [11,12].

Patient symptoms and objective findings are often difficult to reconcile. To treat ONP, a multimodal approach may be beneficial, including the use of ocular surface protection, anti-inflammatory agents, and systemic pharmacotherapy, including tricyclic antidepressants and anticonvulsants [1,13]. However, the best management algorithm is still debated, and evidence-based clinical recommendations for the management of patients with ONP are scarce [1,2].

Given the neuroanatomical and functional structure of the TCC and taking into account that GONB is effective in the treatment of several headache disorders [14,15,16,17], there are insights that suggest inhibiting the GON can alter trigeminal transmission and that the functional connectivity in the TCC can be utilized to manage ONP. We thought that the “convergence” in the TCC might have a role in pain modulation and hypothesized that repeated GONB could reduce ocular pain signals induced by sensitization in the ocular sensory apparatus (Figure 1). We retrospectively analyzed the clinical data of ONP patients who underwent repeated GONB.

## 2. Materials and Methods

### 2.1. Study Design

This study is a single-center retrospective observational study. In this study, we analyzed the medical records of 204 patients who were referred to the pain clinic and subsequently underwent repeated GONB for chronic ONP (>3 months) between January 2008 and February 2022. Approval from the Dong-A University Hospital Institutional Review Board and Research Ethics Committee of Hospital was obtained on 4 May 2022. We registered this study in the Clinical Research Information Service of the Republic of Korea on 26 October 2022. All methods were implemented in accordance with relevant guidelines and regulations in the Declaration of Helsinki.

### 2.2. Study Population

In this single center retrospective study, the data of 204 patients who had received GONB for ONP included sex, age, history of ONP (months), surgical history and trauma, and underlying disease. The underlying cause of ocular pain was first identified through sufficient evaluation, including slit-lamp examination, and then existing ocular surface diseases and nociceptive pain were managed. Ocular surface nociceptive pain management includes lifestyle changes, medications, and adjuvant therapies. Patients were treated with warm compresses and received instruction on eyelid hygiene, nutrition, stress management, and lifestyle changes. Medications for ocular surface pain included artificial tears and lubrication, topical nonsteroidal anti-inflammatory drugs and steroids, autologous serum, and topical cyclosporine A. Adjuvant therapies such as taping eyelids while sleeping, scleral contact lenses, bandage contact lenses, and punctal plugging were applied if needed. Patients whose symptoms persisted even after the underlying causes were conservatively treated for at least 3 months consulted our pain medical center and underwent repeated GONB. The exclusion criteria were as follows: contraindications to occipital nerve block (patients who had refused treatment, severe coagulopathy, infection of the scalp, cervical instability or acute fracture, Arnold-Chiari syndrome, and inability to lie prone).

### 2.3. Intervention

**Figure 2:**
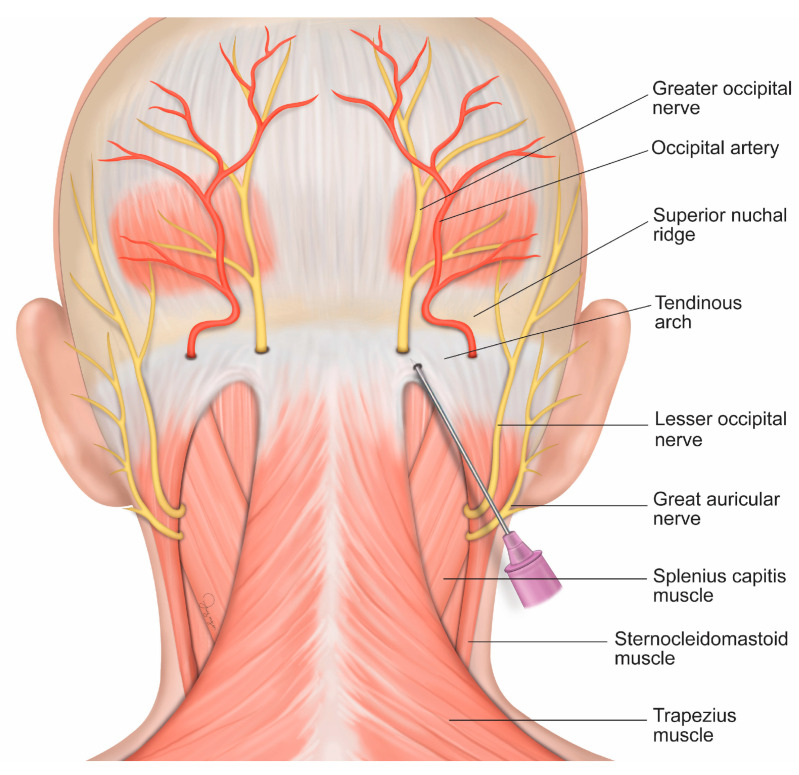
Schematic illustration depicting the injection site for greater occipital nerve block.

Before injection, the patient was placed in a prone position, and a pillow was placed on the chest for neck flexion. A scrub hair cap was used to secure the hair, and the patient’s clothes were taped to expose the neck and occipital area. If no infection or skin lesion was found after the scalp was checked, the hair and scalp were cleaned and prepared using standard skin cleaning and preparation protocols. The hair was not shaved. After preparation of 5 mL of 0.4% lidocaine solution (Huons Co., Seongnam, Republic of Korea), the needle was changed to a 6 cm 23 gauge needle (Koreavaccine Co., Ltd., Seoul, Republic of Korea). Using Trescot’s approach for GONB, the practitioner identified the injection site by placing the thumb at the foramen magnum and the index finger at the conjoined tendon attachment, and identified the injection site at the base of the skull using the middle finger [18]. The needle was directed superiorly and slightly medially until bony skull contact was made (Figure 2). After drawing backward 1–2 mm and gently aspirating the area to avoid intravascular injection, 2 mL of 0.4% lidocaine was injected underneath the conjoined tendon. The procedure was performed on both sides even if the patient felt pain on only one side. The injection area was compressed until bleeding stopped. Then, the patient was placed in a supine position, and a pillow covered with a folded cloth was placed on the neck to compress the injection site. After a 10 min rest, if no adverse events were observed, the session was ended. The injection was performed by a single experienced physician who used the same technique for every patient.

### 2.4. Outcome Measurement

Patients were asked to visit the pain clinic once every two weeks and received GONB up to 10 times. GONB was discontinued if there was no improvement following 3 or more continuous treatments or if the patient refused to continue. Patients continued their previous ophthalmologic treatments while receiving GONB.

ONP can be expressed as various subjective symptoms. In this study, symptoms of ONP were divided into 5 categories: eye pain, dysesthesias/allodynia, non-eye pain, visual disturbance, and tearing. Eye pain was described as stinging, dullness, sharpness, burning, soreness, and irritation. Dysesthesias/allodynia was described as dryness, grittiness, itching, and vague sensation of pressure. Non-eye pain included pain located in the periorbital, frontal, temporal, and occipital areas and cheeks. Visual disturbance and tearing were also included in this study. They occur when some axons from the trigeminal nucleus travel to regions in the brain to adjust lacrimation (superior salivatory nucleus) and blinking (facial motor nucleus) [19].

The primary outcome of this study was the overall pain relief scale (PRS) score after repeated injections. The secondary outcomes were the PRS scores for eye pain, dysesthesias/allodynia, non-eye pain, visual disturbance and tearing, and adverse events. The severity of initial clinical symptoms was measured by the numerical rating scale (NRS), where 0 points indicated no pain and 10 indicated the worst pain imaginable. The post-treatment outcomes were measured using the PRS at the next visit every 2 weeks. For the PRS assessment, the patients were instructed to consider the pain of the first visit as the baseline (10) and then indicate whether pain was alleviated by reporting a lower score [20]. All patients were instructed on using the NRS and the PRS. The NRS and PRS scores are routinely used to assess pain in our medical center. This is because in order to choose a treatment (drugs, injections, etc.), we need to know the severity of the pain and how much it has been reduced. For pain assessment, although the visual analog scale (VAS) is also a common tool used to quantify pain severity, we chose the NRS for pain assessment because using the PRS in combination with the NRS increased the objectivity of the assessment when compared to the VAS [20].

In addition, the baseline patient characteristics, clinical symptoms, and adverse events were compared and analyzed.

### 2.5. Statistical Analysis

The data are presented as frequencies with percentages for categorical variables and means ± standard deviations and medians (IQRs, interquartile ranges) for continuous variables. Considering the unbalanced nature of the repeated measured data, a linear mixed model (LMM) with random intercepts was used to fit a model. The LMM model included repeated measures of numeric variables as dependent variables; group, time, and group × time interaction as fixed effects; and subject as a random effect. To avoid making any assumptions about the covariance structure, we used an unstructured covariance matrix that was allowed to differ across groups for the LMM analysis, and the Bonferroni procedure was applied in post hoc analyses. For graphical visualization, an error bar chart and spaghetti plot with an estimated line according to an LMM are displayed.

All statistical analyses were carried out using SPSS 26.0 (IBM Corp. Released 2019. IBM SPSS Statistics for Windows, Version 26.0. Armonk, NY, USA: IBM Corp) and R version 4.1.3 (Copyright^©^ 2022 The R Foundation for Statistical Computing). *p* values less than 0.05 were considered statistically significant.

## 3. Results

**Figure 3:**
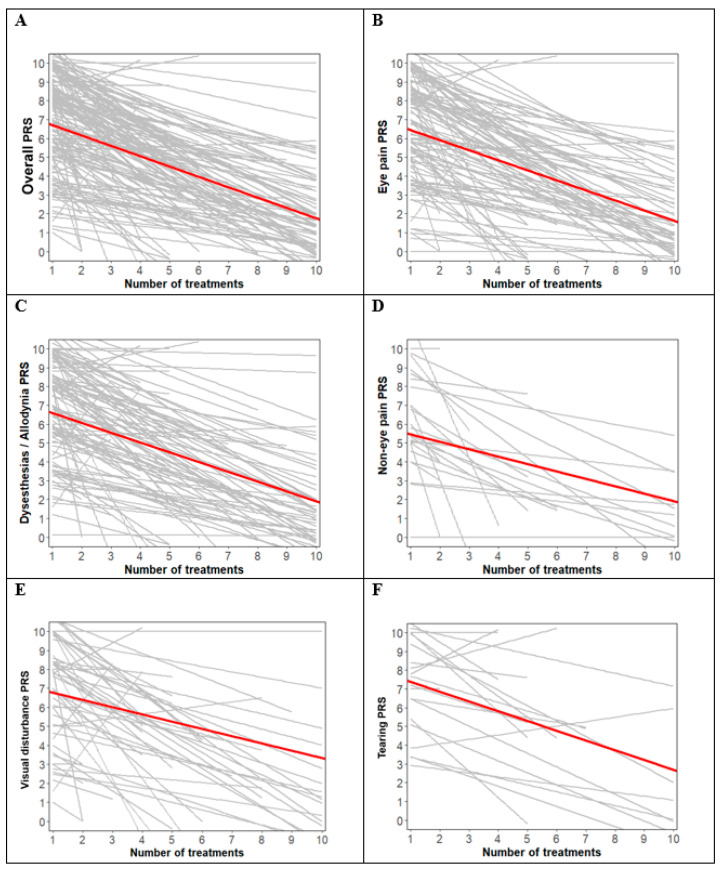
Spaghetti plot with the estimated line according to a linear mixed model. The thin lines represent individuals, and the red bold lines represent estimates of the PRS according to a linear mixed model. (**A**) Overall PRS; (**B**) eye pain PRS; (**C**) dysesthesias/allodynia PRS; (**D**) non-eye pain PRS; (**E**) visual disturbance PRS; (**F**) tearing PRS. PRS (pain relief scale).

The patients’ demographics and clinical characteristics are presented in Table 1. The primary endpoint of the study, the PRS score after GONB, is displayed using a spaghetti plot with an estimated line according to an LMM (Figure 3). The differences and magnitude of decrease in the overall PRS score at the assessment time points were statistically significant (estimate = −0.55, *p* < 0.001). The estimate represents the expected change per unit of treatment. The PRS score for eye pain also significantly decreased during the assessment time points (estimate = −0.54, *p* < 0.001). In addition, we found significant decreases, such as −0.52 in the PRS score for dysesthesia/allodynia (*p* < 0.001), −0.39 for non-eye pain (*p* < 0.001), −0.38 for visual disturbance (*p* < 0.001), and −0.52 for tearing (*p* < 0.001). Adverse events, including the recurrence of ONP, are presented in Table 2. There were 2 patients who had recurrence of ONP and 7 patients who had adverse events (neuritis: 4, transient facial hypoesthesia: 2, skin irritation: 1).

## 4. Discussion

In this study, we aimed to evaluate the effect of GONB on patients who had suffered from ONP for at least 3 months despite conservative treatment. Although many treatments, including systemic pharmacotherapies and lifestyle changes, have been introduced, the management of ONP remains a challenge. Currently, multimodal therapy is the mainstay of treatment due to the various and complex underlying factors of this disease [1,13,19]. Because all the patients in this study had already received traditional treatments for at least 3 months and reported dissatisfaction, we demonstrated that GONB can offer additional pain relief in patients with ONP. According to the results of the study, the differences and magnitude of decrease in the PRS score for all symptoms at the assessment time points were statistically significant (estimate = −0.55, *p* < 0.001) (Figure 3). This suggests that GONB for ONP can reduce symptom severity.

In this study, patients had ONP for more than three months. Chronic pain usually has a neuropathic component and requires multimodal pain treatment. Several treatments, including alpha-2 adrenergic agonists, opioids, serotonergic drugs, and gabapentin, modulate neuropathic pain by affecting the trigeminal nucleus to diminish excitation or increase inhibition pathways [21]. Nonpharmacologic interventions have also been applied in the treatment of ocular pain, such as stellate ganglion block [22]. We used GONB as one of the multimodal pain treatments. According to our study outcome, the PRS scores of the patients who had been administered GONB were reduced. The reason for the relief of clinical symptoms associated with ONP after GONB is not clear. Several hypotheses have been proposed, including the inhibition of central pain mechanisms at the brainstem level. GONB may modulate pain by inhibiting nociceptive input to the TNC, regardless of whether the pain originates from the GON or the trigeminal nerve. Similar to other drugs, GONB may reduce pain by diminishing excitation or increasing inhibition pathways.

This report summarizes the authors’ more than 14 years of experience in treating ONP with greater occipital nerve blocks. This study describes the clinical features of ONP, injection composition, and injection technique. The authors previously treated a patient with occipital pain with GONB, and the patient said that existing chronic eye pain had disappeared. Some headaches that originated from trigeminal activation were relieved in severity after GONB, and this response could be explained only by convergence between the trigeminal nerve and upper cervical nerve [23]. Electrophysiology studies in rats have identified a population of nociceptive neurons located in the C2 spinal dorsal horn that receive input from both the occipital nerve and the ophthalmic branch of the trigeminal nerve [24]. Furthermore, a previous study reported facial hypoesthesia after GONB, providing further clinical evidence for “trigeminocervical convergence” [25]. Accordingly, we considered the treatment mechanism of ONP through GONB and thought that the existing hypothesis of cervical headache, the trigeminocervical convergence mechanism, could be considered a therapeutic hypothesis. Based on this hypothesis, we attempted to treat patients with ONP by collaborating with the ophthalmology department of our hospital, and interesting treatment results were obtained. We treated patients by changing the number of treatments, methods, and medications to make them more effective. After setting up the treatment methods to some extent, we have continued to adapt them to ONP, as requested since 2008. Repeated GONB for ONP treatment has not yet been reported in the literature, and only a few case reports have reported ONP treated via one-time GONB [7].

We only administered a relatively low concentration of lidocaine to affect pain modulation in the TCC. According to previous studies, 0.5% lidocaine can produce sensory blockade, and 0.25% lidocaine can be used for sympathetic blockade [26]. Therefore, to minimize patient discomfort but to affect neuronal function, a concentration of 0.4% lidocaine was selected, although 1–2% lidocaine is usually chosen for peripheral nerve block [27]. We also did not use any steroids, as the goal of the study was not to reduce inflammation. In addition, Ashkenazi et al. [28] demonstrated that GONB using local anesthetics with triamcinolone could not improve the outcome of migraine compared to local anesthetics alone. As we did not use any steroids, complications related to steroids, including flushing and alopecia, were not observed. No other adverse events, such as dizziness and nausea, were detected. Four patients reported pain in the injection site, which was suggestive of greater occipital neuritis and easily managed at the next visit after adding a small amount of triamcinolone to the lidocaine solution.

Additionally, as secondary sensory neurons in the TNC can receive ipsilateral and contralateral inputs from the GON, we performed GONB on both sides even if the patient reported pain on only one side because a blockade on one side could affect the contralateral side [29].

The present study has several limitations. First, since no widely accepted quantitative assessment of ONP exists in the literature, we had no choice but to rely on the subjective evaluation of patients, the NRS and the PRS. However, according to the Assessment Committee of the Neuropathic Pain Special Interest Group guidelines on neuropathic pain assessment, the intensity of pain should be assessed with the NRS, and the PRS is one of the tools recommended for assessing the effect of treatment for neuropathic pain [30]. Second, the study design was not placebo-controlled. It was fundamentally difficult to set up a placebo group due to the block’s invasiveness and ethical issues. Thus, further studies are needed to establish the effects of GONB in an equivalent control group. Despite the lack of a comparison group, when the number of time points was increased, the performance of many of the longitudinal methods also improved [31]. Long-term longitudinal studies in which up to or more than eight time points per patient are included are meaningful considering that currently, relatively few time points are typical in biomedical studies. Longitudinal methods provide a more comprehensive research perspective that allows a better understanding of the degree and direction of change over time. Additionally, observational and single-armed studies are worth considering when very few studies have been performed and any “active treatment” is known, and patients may be reluctant to agree to random assignment considering the invasive nature of these interventions. Third, GONB was performed using a conventional landmark-guided technique. In this study, a single experienced anesthesiologist performed GONB using the same technique. Kissoon et al. [32] demonstrated that landmark-guided GONB was not significantly different in terms of changes in the NRS score after 2 weeks and the incidence of adverse events when compared to ultrasound-guided GONB. Fourth, we could not evaluate the rate of long-term recurrence or disease progression because we no longer followed up with the patients after the treatment ended. However, progress was observed for up to 20 weeks, which is clinically meaningful and is a relatively long-term period compared to a previous study of GONB [26,33]. We instructed all patients to visit the clinic again when ONP recurred, and two patients revisited the clinic for recurrence. One patient received 4 more injections, and the PRS score was reduced to 1 again; the other patient received 10 more injections, and the PRS score was reduced to 0.

## 5. Conclusions

This study supports the hypothesis that a series of GONBs may reduce the PRS score and may have therapeutic value in the treatment of patients with ONP. Repeated GONB can be considered a multimodal ONP management method. A multicenter study with a control group will be needed for further evaluation of GONB’s effect for ONP.

## Figures and Tables

**Table 1:** Demographics and clinical characteristics.

	All Patients (*n* = 204)
Sex	
Male	28 (13.7)
Female	176 (86.3)
Age, years	62.50 (55.00–71.00)
Height, cm	158.00 (152.00–162.00)
Weight, kg	58.50 (50.00–62.00)
Body Mass Index, kg/m^2^	23.02 (21.06–24.85)
History of ONP (months)	12.00 (3.00–36.00)
Surgical history & trauma	
Cataract surgery	76 (37.3)
Refractive surgery	25 (12.3)
Eyelid surgery	26 (12.7)
Other eye surgery	2 (1.0)
Trauma	3 (1.5)
Underlying eye diseases	
Dry eye	131 (64.2)
Keratitis	36 (17.6)
Corneal ulcer	7 (3.4)
Conjunctivitis	61 (29.9)
Blepharitis	26 (12.7)
Uveitis	3 (1.5)
Tumor	1 (0.5)
Underlying systemic diseases	
Diabetes	14 (6.9)
Fibromyalgia	2 (1.0)
Depression	5 (2.5)
Sjögren’s syndrome	5 (2.5)
Symptoms	
Eye pain	147 (72.1)
Dysesthesia/Allodynia	138 (67.6)
Noneye pain	26 (12.7)
Visual disturbance	67 (32.8)
Tearing	18 (8.8)
Initial NRS (overall)	5.00 (4.00–7.00)
Initial NRS (eye pain)	5.00 (4.00–8.00)
Initial NRS (dysesthesias/allodynia)	5.00 (4.00–7.00)
Initial NRS (noneye pain)	6.00 (4.00–8.00)
Initial NRS (visual disturbance)	5.00 (4.00–6.00)
Initial NRS (tearing)	4.00 (3.00–6.50)

Variables are presented as medians (25th–75th percentile) for numeric variables and numbers (%) for categorical variables. ONP (ocular neuropathic pain), NRS (numeric rating scale).

**Table 2:** GONB-related complications.

	All Patients (*n* = 204)
Recurrence	2 (1.0)
Adverse events	
Neuritis	4 (2.0)
Infection	0 (0.0)
Transient facial hypoesthesia	2 (1.0)
Dizziness	0 (0.0)
Nausea	0 (0.0)
Skin irritation	1 (0.5)

Variables are presented as numbers (%).

## Data Availability

The data presented in this study are available upon reasonable request from the corresponding author.
